# Recruitment strategies used to enrol healthy volunteers in the first pneumococcal human infection study in Africa: Lessons from Blantyre, Malawi

**DOI:** 10.12688/wellcomeopenres.19271.2

**Published:** 2025-02-26

**Authors:** Edna Nsomba, Anthony E. Chirwa, Clara Ngoliwa, Vitumbiko Nkhoma, Pemphero Liwonde, Edward Mangani, Modesta Reuben, Lorensio Chimgoneko, Lumbani Makhaza, Evaristar Kudowa, Marc Y R Henrion, Neema Toto, Stephen B. Gordon, Dingase Dula, Malawi Accelerated Research in Vaccines, Experimental Laboratory Systems (MARVELS) consortium

**Affiliations:** 1 Clinical and Experimental Medicine, Malawi-Liverpool-Wellcome Trust Clinical Research Programme, Blantyre, Southern Region, P.O. Box 30096, Malawi; 2 Department of Medicine, Queen Elizabeth Central Hospital, Blantyre, Southern Region, P.O. Box 95, Malawi; 3 Department of Clinical Sciences, Liverpool School of Tropical Medicine, Liverpool, L3 5QA, UK

**Keywords:** Controlled Human Infection Model, Streptococcus pneumoniae, Carriage, Conjugate Vaccine, Clinical trial

## Abstract

**Background:**

Human Infection Studies (HIS) also known as Controlled Human Infection Models (CHIM) are a relatively new concept in African countries to clinicians, scientists, and communities alike. We have introduced HIS/CHIM studies to Malawi during the last four years by developing an experimental human pneumococcal carriage model. This CHIM was used to test the efficacy of a licensed 13-valent Pneumococcal Conjugate Vaccine (PCV13) against experimental nasal pneumococcal carriage. Traditional and digital recruitment strategies into this novel trial were explored.

**Objectives:**

To describe various methods of recruitment in this first CHIM study in Malawi.

**Methods:**

The clinical trial within the context of which these data were recorded was registered with the Pan African Clinical Trials Registry (REF: PACTR202008503507113) on 03 August 2020. The project was conducted at the Malawi Liverpool Wellcome Programme (MLW) in Blantyre, Malawi between April 2021, and September 2022. Source populations were college students and community members within Blantyre. Recruitment strategies included sharing study information in written or visual form, community sensitization meetings, snowball contacts (word of mouth from previous volunteers), branded clothing and participating in radio and television programs.

**Results:**

299 volunteers attended screening clinic, of whom 278 were recruited. Sixty-six recruited volunteers (23.7%) were college students and 212 (76.3%) were from the community. Snowball word-of-mouth contacting was the most successful recruitment strategy, with 201 (72.3%) participants recruited using this method. 195 (70.1%) were men of whom 149 (76.4%) joined the study through snowballing.

**Conclusion or recommendation:**

Using a variety of recruitment strategies led to successful recruitment in this novel controlled human infection study. Most participants were recruited through snowballing.

## Introduction

### Rationale

Human Infection Studies (HIS), also known as Controlled Human Infection Models (CHIM), involve introducing a pathogen to a healthy individual under carefully monitored conditions. These studies continue to make important contributions to prevention and treatment of many infectious diseases by offering researchers a platform to evaluate the efficacy of vaccines in protection against infections as well as the immune response generated. The study of vaccines for malaria and influenza have been significantly advanced by CHIM studies ^1^. Following a scale up in production, CHIM studies are now possible at a very much greater scale ^2^. High Income Countries (HIC) have utilized these studies to understand biology and develop vaccines for pathogens of clinical and population health importance such as human influenza viruses, respiratory syncytial virus, severe acute respiratory syndrome coronavirus 2 (SARS-CoV-2), Staphylococcus and Neisseria species. High literacy levels, equitable access to health care, advanced technology, superior physical and digital health services infrastructure, and high socio-economic status have facilitated the success of these novel studies in HICs.

In Africa, not so many countries have conducted CHIM studies so far. Malawi, Kenya, Uganda, and Tanzania are four countries that have established HIS in Africa (Malawi: *Streptococcus pneumoniae* challenge ^3^, Kenya ^4^: *Plasmodium falciparum* challenge, Uganda: Schistosoma challenge ^5^). One of the contribution of Kenyan Malaria CHIM study was that it allowed for an early assessment of vaccine efficacy in a population with the same generation background as the eventual target population. This study built a strong and grounded understanding of the ethics of research used in CHIM models which has helped to inform the future of CHIM models in similar settings. Current CHIM studies in other African countries continue to build on these efforts ^6^.

Other countries such as Zambia ^7^, are following with other study pathogens, and priming their populations for introduction of these novel studies ^7^. Typically, HIS are conducted first in a HIC and after the technology, standard operating procedures and safety have been established, transferred to a collaborating Low-Income Country (LIC). For example, pneumococcal human challenge trials were conducted in Liverpool for a decade following which technology and standard operating procedures were transferred to Malawi for feasibility testing ^8^. Similarly, a study of blood-stage controlled human Plasmodium *falciparum* malaria infection that is ongoing in Tanzania, originated from Oxford University ^9^. As CHIM studies expand and clinical researchers continue to gain more experience, other population at risk have been included. Recently, in Malawi a CHIM study in people living with HIV (PLHIV) was implemented using the same experimental design well-established at the Malawi-Liverpool Wellcome Programme and at Liverpool School of Tropical Medicine ^10^. This has laid a foundation for future at-risk population CHIM models in similar settings.

The first workshop to be convened on human challenge work in Malawi by a technical working group, which included the authors, met in 2017 ^11^. Clinicians, scientists, ethicists, and community leaders discussed the potential benefits of human infection studies (accelerated vaccine development, capacity building) and risks (safety, acceptability, ethical concerns) ^11^. The workshop report highlighted: excellent international clinical standards, local capacity building and ownership, a rigorous informed consent process, mitigation of challenges with transport and access to health facilities, appropriate economic compensation, and managing community and media perceptions as key issues to address to ensure success of human infection studies in a setting like Malawi ^11^.

Next, the researchers conducted a study exploring acceptability of human infection studies using focus groups and key informant interviews with Blantyre-based research staff, medical students, and community representatives, clinicians, ethics committee members, and district health government officials ^11^. Overall, HIS studies were favourably perceived and potentially beneficial provided the following conditions were met; voluntary and informed consent, rigorous inclusion/exclusion criteria, provision of medical check-ups and monitoring, appropriate compensation, and robust community engagement ^11^.

Acceptability work paved way for feasibility testing of the human challenge model among 24 healthy volunteers, whose experiences with the trial from recruitment methods, compensation, inoculation with live bacteria, study procedures (nasosorption, nasal scrape with rhino probe, nasal wash, throat swab, saliva collection quarantine and residential stay post-challenge, were sought after trial completion ^12^. Motivation for joining the study despite initial reservations included altruism, patriotism, and monetary gains ^12^. Although the participants did not experience adverse events in the short duration of the study (21 days) they were concerned about future unanticipated risks ^12^. The volunteers admitted that the concept of human challenge trials was completely novel and recommended extending information and education about the model to the wider Malawian population ^12^.

In the present paper, we discuss experiences and lessons we have learnt about recruitment through the process of scaling up from a feasibility study in tens of participants to a randomized controlled vaccine trial requiring screening of more than 250 participants.

### Objectives

We describe methods used in recruiting participants in a pneumococcal CHIM study in Blantyre Malawi and highlight lessons learned in the process. The objective of the pneumococcal CHIM study was to test the efficacy of a licensed 13-valent Pneumococcal Conjugate Vaccine (PCV-13) against experimental nasal pneumococcal carriage.

***Study site.*** This study was conducted in Blantyre, southern region of Malawi among health participants who took part in the first Controlled Human Infection Model study to assess the efficacy of PCV-13 vaccine.

***Study Population.*** The efficacy of Pneumococcal Conjugate Vaccine (PCV-13) against experimental nasal pneumococcal carriage study recruited healthy participants aged between 18–40 years from surrounding communities and colleges. The study protocol and results can be accessed on https://wellcomeopenresearch.org/articles/6-240 and https://www.thelancet.com/journals/lanmic/article/PIIS2666-5247(23)00178-7/fulltext respectively.

## Methods

### Trial design

This was a qualitative description of recruitment methods used to recruit participants in a double-blinded, parallel-arm, randomized controlled trial investigating the efficacy of PCV13 or placebo (allocation ratio PCV13: placebo 1:1) against experimental pneumococcal carriage of *Streptococcus pneumoniae* serotype 6B (SPN6B). The study protocol has been published in Wellcome Research Open.

## Methods

### Data collection and analysis

The information in this study was collected retrospectively by calling all participants who consented and participated in the pneumococcal CHIM study. This was because in the main study (PCV-13 study) participants’ information on how they heard about the study was not collected and documented. The main study was approved by ethics committee. Participants were asked to recall how they heard about Pneumococcal CHIM study and this information was documented on an excel sheet. We analyzed the data by counting each category of the planned sensitization methods.

*Sensitization meetings*: Sensitization meetings were conducted at eight surrounding colleges in Blantyre namely Malawi College of Health Sciences, Malawi University of Business and Applied Sciences, formerly The Polytechnic, Kamuzu University of Health Sciences, Malawi Institute of Journalism and Malawi Institute of Tourism. Each sensitization meeting was attended by approximately 50 students. Additional sensitization meetings were conducted at the Malawi-Liverpool-Wellcome Programme main site and invited research and clinical staff. Individuals interested in the study provided their telephone numbers to the study staff and verbally consented to be phoned to schedule an information session at the clinic. Sensitization meetings were interrupted from July to August 2021 by school closures due to the fourth COVID wave but resumed in September 2021.

*Snowball recruitment*: Snowball recruitment or sampling, also called chain-referral sampling, is an informal spread of study-related information to potential participants by word of mouth ^13^. Snowball recruitment in this study occurred naturally without study staff influence. Potential participants and existing participants shared study information with their peers, who came to volunteer for the study as a result.

*Radio and Television*: While initially the study team was careful to perform only targeted sensitization during the feasibility study, the proven safety of the model demonstrated for over three years provided later confidence to expand awareness of human infection studies to the wider community. In May 2022, four weekly radio broadcasts about human infection studies and the PCV13 study were conducted by study clinic and laboratory team members. The broadcasts were one hour long, with a text and phone dial in segments for listener engagement. These broadcasts were all live and recorded in both English and Chichewa. There was very high engagement from the listeners during the radio programmes. Similarly, a television programme was recorded with a live studio audience and aired twice in June 2022. The live audience engaged well with the study team and asked relevant questions.

*Digital media*: The study team recorded a video for MLW’s YouTube channel describing the importance of human infection studies in Africa and detailing the PCV13 trial. In addition, a digital study flyer was circulated on WhatsApp.

*Increased visibility*: The study team utilized branded clothing for participants and staff members to increase visibility and generate interest about the study.

*Clinic recruitment:* Recruitment to the pneumococcal CHIM study itself was as follows. Potential participants who showed interest were invited via telephone to an in-person information visit (visit A) to the research clinic. The information visits were conducted in groups and lasted approximately an hour. During this visit, A study nurse or clinician provided detailed information about the study including screening, vaccination, inoculation, quarantine, and safety procedures and follow up. Risks were discussed in detail. Materials used to collect samples were also demonstrated. At this stage, participants did not require to disclose whether they will join the study or not but were encouraged to think about it and to decide later. At the end of the visit, the potential participant’s information, including their name, contact number, age, sex, residence, and their information sources regarding the study were recorded. Information sources included college sensitization campaigns, snowballing, adverts, social media and digital programs on radio and television. In addition, information to define the participants’ community category was collected. They were defined as college students or not a college student (referred to as ‘the community’).

During a second visit intended to obtain individual consent (visit B), data were collected to show how many participants from each category showed interest in joining the study and how many of each category were both eligible and were successfully recruited, consented and vaccinated.

**Figure 1.**
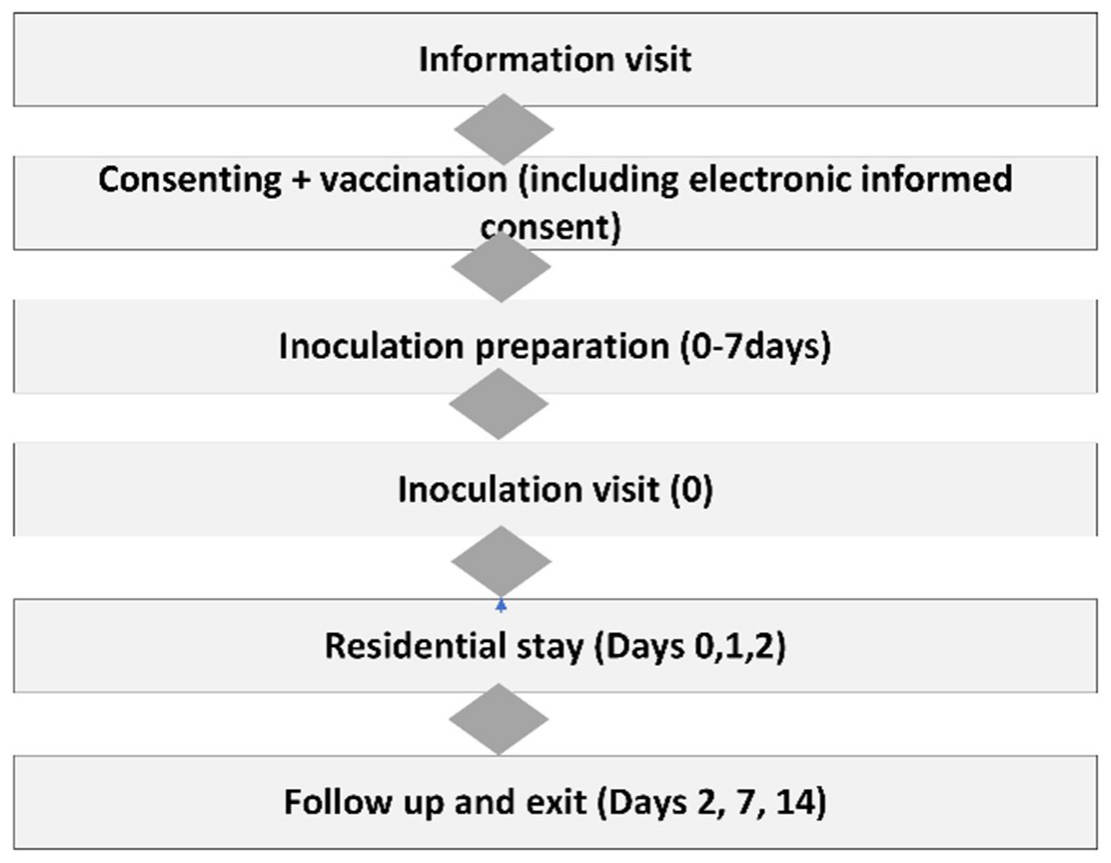
Summary of recruitment and follow up procedures in the main trial; PCV13 trial. This figure is an original figure produced by the author(s) for this review article.

Following screening and recruitment, participants underwent randomization, vaccination, inoculation and follow up including residential stay and exited the study. Study procedures are summarized in Figure 1.

## Ethical approvals

The CHIM study including recruitment strategies was approved in Malawi by the National Health Sciences Research Committee on 1 May 2020 (REF: 16/07/2519) and Pharmacy Medicines and Regulatory Authority (REF: PMRA/CTRC/III/10062020121) and in the United Kingdom by the Liverpool School of Tropical Medicine on 23 April 2021 (REF: 20-021). The trial was registered with the Pan African Clinical Trials Registry (REF: PACTR202008503507113) and can be found on https://pactr.samrc.ac.za/TrialDisplay.aspx?TrialID=12124.

## Results

A total of 299 participants were screened for the study, of which 278 were recruited. 209 (69.9% and 195 (70.1%) of the screened and recruited volunteers, respectively, were males.

### Screening and recruitment by recruitment strategy

*Snowballing:* 215 of 299 (71.9%) screened indicated that they were motivated to join through snowball recruitment. Of these, 201 were eventually enrolled in the study. 21 volunteers were excluded for not meeting one or more of the inclusion criteria ( Table 1).

**Table 1.** Inclusion and Exclusion Criteria.

Inclusion criteria	Exclusion criteria
• Adults aged 18–40 years • Fluent spoken and written Chichewa or English • Own a cell phone	• Previous pneumococcal vaccination • HIV-infection seropositive • Close physical contact at-risk individuals • Allergy to penicillin/amoxicillin • Acute illness • Chronic illness that may impair immune response or impair ability to comply with study procedures and safety • Pregnancy • History of drug or alcohol abuse • History of Smoking • Unable to give informed consent • Participant is positive for *Streptococcus* *pneumoniae* serotype 6B

*Sensitization:* 82 of 299 (21.4%) screened were motivated after a sensitization event ( Table 2a and Table 2b).

**Table 2a.** Screening methods by sex.

	Total screened (N=299)	Snowball n (%)	Sensitization n (%)	Radio and television n (%)	Poster n (%)
Male	209	160 (76.6)	48 (23.0)	1 (0.4)	0 (0.0)
Female	90	55 (61.1)	34 (37.8)	0 (0.0)	1 (1.1)

**Table 2b.** Recruitment methods by sex.

	Total recruited (N=278)	Snowball n (%)	Sensitization n (%)	Radio and television n (%)	Poster n (%)
Male	195	149 (76.4)	45 (23.1)	1 (0.5)	0 (0.0)
Female	83	52 (62.7)	30 (36.1)	0 (0.0)	1 (1.2)

*Radio and television:* This strategy motivated only one individual to screen and enrol in the study ( Table 2a and Table 2b).

*Poster:* Only one individual was screened and enrolled after seeing a study poster ( Table 2a and Table 2b).

### Participant feedback on exiting the PCV13 study

213 participants accepted to be interviewed regarding their experience in a CHIM at study exit. Responses were recorded using a Likert scale survey. The majority either strongly agreed or agreed that study processes from informed consent, through recruitment, safety monitoring, compensation and quarantine-were a positive experience. 95.3% stated that they would recommend study participation to a friend ( Table 3).

**Table 3.** Participant feedback at study exit.

Column1	Strongly agree n(%)	Agree n(%)	Neutral n(%)	Disagree n(%)	Strongly diasgree n(%)
The approach used for study recruitment was appropriate	146(68.5)	62(29.1)	5(2.3)	0	0
The information provided before consenting was appropriate	155(72.8)	58(27.2)	0	0	0
The medical questions and tests used before consenting were appropriate	142(66.7)	66(31.0)	5(2.3)	0	0
There was sufficient time to consider the study before consenting	147(69.0)	60(28.2)	5(2.3)	1(0.5)	0
The finger print scanner was acceptable way to confirm my identification	164(77.0)	46(21.6)	3(1.4)	0	0
The clinical team treated you with respect and kindness.	173(81.2)	39(18.3)	1(0.5)	0	0
The safety monitoring procedures for the study were appropriate	151(70.9)	59(27.7)	3(1.4)	0	0
The study follow up procedures did not cause inconvenience	122(57.3)	73(34.3)	14(6.6)	4(1.9)	0
The accommodation provided after inoculation was satisfactory	146(68.5)	54(25.4)	11(5.2)	2(0.9)	0
The meals provided at the accommodation were satisfactory	155(72.8)	49(23.0)	8(3.8)	1(0.5)	0
The location of the accommodation was convenient	137(64.3)	65(30.5)	8(3.8)	3(1.4)	0
The compensation provided by the study was appropriate	99(46.5)	72(33.8)	34(16.0)	5(2.3)	3(1.4)
I would recommend participant in this study to a friend	142(66.7)	63(29.6)	7(3.3)	1(0.5)	0

## Discussion

In this article, we describe recruitment methods utilized in a pneumococcal human infection study. We sought to detail the contribution of various recruitment strategies to recruit and enroll healthy participants in a CHIM study in Blantyre, Malawi. Among the recruitment strategies used in this study, an overwhelming number of participants were recruited via snowballing or word of mouth networking. 215 out of 299 participants who were screened representing 71.9% joined through snowball method. Out of these 201 were enrolled in the study. The higher enrolment efficiency of snowballing was because study related information was widely shared to potential study participants prior to information visit at the clinic. The advantages of prior sharing of information relieved potential participants of stress and anxiety because of the assurance from other participants who had passed through the same process. Snowballing strategy reduced the number of community sensitization meetings because the study had enough number of people who have attended information visit way before the target period.

Snowballing is often more cost-effective than other recruitment methods, particularly when traditional advertising like posters, using television or radio are costly or unavailable. The snowball method reduced the need for large-scale recruitment campaigns because the participants did much of the recruitment themselves.

PCV-13 study had a good participant retention and snowball recruitment method partly contributed to it. Evidence shows that because participants are recruited through referrals from people they trust, such as friends and family, they may be more likely to engage and remain committed to the clinical trial ^14^.

The drawback to snowballing recruitment was that participants recommended the study to relatives and friends from the same location and with similar characteristics ^15^. This has the potential to over-represent specific social and demographic group. This let to other participants from other location from hearing about the study. This is the first study of its kind of which we are aware. The strengths of this study are that several institutions and potential volunteer groups were observed, and many methods of recruitment were attempted. Some of the limitations are that not all of the recruitment processes and decision making can be observed and we could not explore further on the snowball method. Also, it can be difficult to ascertain the accuracy of the information that participants share with others.

We suggest that hearing about the study from a former or current volunteer may reinforce trust in the safety of the study in potential volunteers and encourage them to join. There has been evidence suggesting this in participant studies in Malawi and in Kenya. We also suggest that in future CHIM studies, it is very important to begin with community engagement activities and adopt snowballing as the main strategy.

On reflection, we consider that the tools used by the study team to inform and educate the community about the study may not have been well understood or accepted by the targeted audience and may need to be reviewed. In a different view, a malaria controlled human infection study conducted in Kenya, community engagement facilitated understanding of the study process, especially in participants with low levels of education ^16^.

While radio and television, done in both English and Chichewa, attracted an audience and active participation, this did not directly translate to an increased number of volunteers. The suitability of the messaging tools needs further exploration. Engaging former and current volunteers to participate in study sensitization activities may need to be considered.

Our study recruited more male than female volunteers. Possible reasons for this could be greater autonomy and decision-making power regarding consent to research participation among men than women in Malawi, although this was not formally explored in this study. Another reason could be that the information visits reached more men than women and via snowballing they predominantly enlisted their friends, mostly men too. Thirdly, if the study was viewed as risky, males may have had a greater risk tolerance. Targeting women through women church groups and community village banks might increase participation of women in CHIM studies in Malawi.

## Conclusions

In conclusion, engaging current and former volunteers in novel trials like human infection studies is a possible strategy that can encourage community acceptance and participation in settings like Malawi. It is very important that researchers must consider snowballing limitations carefully and take steps to mitigate them such as employing strategies for a diversified sample. More work needs to be done to explore how increased participation from women can be ensured.

## Data Availability

Figshare: Underlying data for ‘Recruitment methods and participant experiences in the first controlled human infection study in Blantyre, Malawi.’ https://doi.org/10.6084/m9.figshare.22567513.v1 This project contains the following underlying data: Data file 1. (The attached file contains the following information: VisitA_month: Date of information visit, VisitB_date: Screening visit, Age, Sex, Recruitment and, Vaccination status, Study completion status, Column I to U represents Likert scale of participant experiences with summary of findings in Table 3 in this article.) Figshare: Extended data for ‘Recruitment methods and participant experiences in the first controlled human infection study in Blantyre, Malawi.’ https://doi.org/10.6084/m9.figshare.22567513.v1 This project contains the following extended data: Data file 1 (Description of data.) Data are available under the terms of the Creative Commons Zero “No rights reserved” data waiver (CC0 1.0 Public domain dedication).

## Software availability

Data was collected electronically using Open Data Kit (ODK) on an Android device. To complement ODK functionality, an additional in-house application was used called ODK lookup updater application, which helped to enforce data validation at the point of data collection.

Data was validated at the point of entry using field restrictions embedded within the form to avoid collection of invalid and out of range data for numeric fields. Skip logics were also built into the form to eliminate collection of irrelevant or redundant data. Form level calculations were used to evaluate and validate data like eligibility criteria to avoid human error in decision making for such critical study decisions. And finally, an inhouse application was used for cross form verification of previously collected critical participant information.
