# Supplementary material for: Recruitment strategies used to enrol healthy volunteers in the first pneumococcal human infection study in Africa: Lessons from Blantyre, Malawi
Source: Wellcome Open Res. 2025 Feb 26;9:216. Originally published 2024 Apr 24. [Version 2] doi: 10.12688/wellcomeopenres.19271.2 (PMC11971628; doi:10.12688/wellcomeopenres.19271.2)
Supplement: Peer review history

# Reviewer report: Reviewer response for version 2

**DOI:** 10.21956/wellcomeopenres.26260.r120636

**Published:** 2025-04-04

**Authors:** Patrick Harris

**Affiliations:** 1 UQ Centre for Clinical Research, University of Queensland, Queensland, Australia

Human challenge studies are a relatively new concept in the African context, yet understanding the effect of new interventions in these populations is important. Hence this study adds to our understanding of how these studies can be effectively delivered in this setting.   The study presents meaningful data and appears to have been well conducted . The data are available and the conclusions are justified  - although the results are largely descriptive. The finding that snowballing was the moist efficient strategy for enrolment is notable. 

 Specific comment:

 - The term "sensitization" is used throughout, but the exact process is not described in detail. Some additional information on how this was done would be useful

Is the work clearly and accurately presented and does it cite the current literature?

Yes

If applicable, is the statistical analysis and its interpretation appropriate?

Not applicable

Are all the source data underlying the results available to ensure full reproducibility?

Yes

Is the study design appropriate and is the work technically sound?

Yes

Are the conclusions drawn adequately supported by the results?

Yes

Are sufficient details of methods and analysis provided to allow replication by others?

Yes

Reviewer Expertise:

AMR research, trials, genomics

I confirm that I have read this submission and believe that I have an appropriate level of expertise to confirm that it is of an acceptable scientific standard, however I have significant reservations, as outlined above.

# Reviewer report: Reviewer response for version 2

**DOI:** 10.21956/wellcomeopenres.26260.r119636

**Published:** 2025-04-02

**Authors:** Dorcas M Kamuya

**Affiliations:** 1 Kenya Medical Research Institute (KEMRI) - Wellcome Trust Research Programme, Kilifi, Kenya

Recruitment strategies for research participants in clinical trials and in controlled human infection studies/models (CHIM) in particular is an area of great interest in bioethics and engagement discourses. A description of effective strategies for controlled human infection studies would provide much needed information for sites that are considering these types of studies, and highlights the important contribution that this publication would make. However, there are some major revisions that would need to be addressed if the work described is to meet the criteria for quality and rigorous qualitative research

 1. **Background sectio**n: whilst it provides good information about the 1sy pneumococcal CHIM study in Malawi and previous work, it does not at all highlight the issue of recruitment strategies in CHIM (or even in clinical trials or research studies), and why that is an area of interest for the current research.  It is thus not clear why recruitment is a topic of interest in this manuscript.

 2. **Methods**: Following above, the entire methods section is about the methods that were used in the main pneumococcal CHIM study.; only one paragraph/sentence mentions that the qualitative study recruited participants from the main study. This methods section should be about the qualitative study methods and analysis approach and should include information such as a) the recruitment methods that was used in the qualitative used b) justification for the respondents that were recruited, for example, why did the qualitative study also not include those that participated in sensitization meetings but did not participate in the CHMI study ie why did it limit itself to only those that participated in the CHMI study? c) why were exit interviews an appropriate method for the qualitative research? what informed the choice of this method? and what is its limitation d) Likert scale questions were used, why was this chosen yet there is criticism of using likert scale questions especially in LMICs e) what approach (in person? telephone interviews?) and who collected the data? f) how was data quality counterchecked?

 3. Data analysis: How was the qualitative data analysed?  what variables were considered and what informed the selection of those variables?

 4. *NB: Qualitative research requires transparency about the entire research (which assists in assessing the quality/rigour of the research and of the reported results), but the current manuscript seems not to address many of these (please check the criteria for reporting qualitative research that is included in guidance of most journals including  WellcomeOpen)

 5. Reflexivity and positionality of the researchers is missing - this is part of being transparent and includes explicitly descriptions of any researcher biases and how these were addressed across the qualitative research cycle.

 6. Since description provided in the methods section  relate to the recruitment methods of the main CHIM study(and not of the qualitative research), I suggest that all that information is moved to the results section. The methods section should then be a description of the qualitative research methods (as alluded in 2 above)

 7. **Ethics review** - the approval described in this research seems to be that of the main CHMI study, what about the qualitative research of it? Kindly clarify and also describe how consent was sought for the qualitative research.

 8: **Results section:** Here present the methods that were used to recruit participants in the main study and information about what informed the choice. Given that several strategies, including expensive one such as radio were used, why was a plethora of strategies considered?

 9. Were there participants who reported participating in several recruitment strategies? how was this handled in analysis?

 10. The participant feedback at exit seems to be highly positive, is there a reflection of a) how social desirability might have influenced responses and link back to researcher positionality b) whether this method of collecting data was the most appropriate -ie a critique of the method since on other settings narratives have been used to describe experiences of participating in CHMI studies 

 11. **Discussion**: it would be important to discuss the findings with references to some of the ethical and social science theories e.g. snowballing and social capital, trust, network analysis and what informs us about what was going on etc.

 12.  Also some information on what sort of information was shared by those that recruited others through snowballing - for example were some over-emphasize on the benefits and not the risks of participation? 

 13. A reflection of what ' *effective recruitment strategy'* means would be very helpful. Meeting target numbers is not the same as effective recruitment, particularly if people do not know that they are in researcher/trial and what is involved in the research. 

 14: The *ethics of using current and former research participants to recruit in studies* - also need to be articulated, both the positive and the negative implications and how these can be balanced.

Is the work clearly and accurately presented and does it cite the current literature?

Partly

If applicable, is the statistical analysis and its interpretation appropriate?

Not applicable

Are all the source data underlying the results available to ensure full reproducibility?

Yes

Is the study design appropriate and is the work technically sound?

Partly

Are the conclusions drawn adequately supported by the results?

Partly

Are sufficient details of methods and analysis provided to allow replication by others?

No

Reviewer Expertise:

Bioethics, ethical issues in Human Infection studies, community engagement

I confirm that I have read this submission and believe that I have an appropriate level of expertise to confirm that it is of an acceptable scientific standard, however I have significant reservations, as outlined above.

# Reviewer report: Reviewer response for version 2

**DOI:** 10.21956/wellcomeopenres.26260.r119635

**Published:** 2025-03-31

**Authors:** Oren Ziv

**Affiliations:** 1 Soroka University Medical Center, Ben-Gurion University of the Negev, Beer-Sheva, Israel

The authors have made the necessary changes to the paper. I have no more comments. Good work.

Is the work clearly and accurately presented and does it cite the current literature?

Yes

If applicable, is the statistical analysis and its interpretation appropriate?

Yes

Are all the source data underlying the results available to ensure full reproducibility?

Yes

Is the study design appropriate and is the work technically sound?

Yes

Are the conclusions drawn adequately supported by the results?

Yes

Are sufficient details of methods and analysis provided to allow replication by others?

Yes

Reviewer Expertise:

otolaryngology, epidimiology, PCV's

I confirm that I have read this submission and believe that I have an appropriate level of expertise to confirm that it is of an acceptable scientific standard.

# Reviewer report: Reviewer response for version 1

**DOI:** 10.21956/wellcomeopenres.21354.r82654

**Published:** 2024-06-12

**Authors:** Oren Ziv

**Affiliations:** 1 Soroka University Medical Center, Ben-Gurion University of the Negev, Beer-Sheva, Israel

The article discusses Human Infection Studies (HIS) in low-income countries(LICs), focusing on their implementation in Malawi, Kenya, Uganda, and Tanzania. HIS involve introducing a pathogen to a healthy individual under controlled conditions to study disease and develop treatments. The paper details recruitment methods for a pneumococcal HIS in Malawi, including sensitization meetings, snowball recruitment, and media campaigns. It highlights the success of snowball recruitment and identifies challenges such as the need for better-targeted messaging and increased female participation. Ethical approvals and participant feedback are also addressed, with most participants having a positive experience and recommending the study to others.

 Strengths

 1.     Comprehensive Recruitment Methods.

 2. Feedback: High levels of participant satisfaction and willingness to recommend the study suggest good management and ethical conduct.

 3. Pioneering Study in Malawi: This study sets a precedent for conducting HIS in Malawi, contributing valuable insights for future research in LICs.

 Weaknesses

 1. Limited numbers of participants.

 2. The study recruited significantly more males than females, which can be a confunder.

 Overall, it is a very nice paper, written well, and designed well.

 Minor revisions to be made:  

 1. Need to add a section that should address the study's limitations (number of participants, time extra).

 2. In the discussion there need to be a compression of the results in this study to other countries in which similar studies were conducted.

Is the work clearly and accurately presented and does it cite the current literature?

Yes

If applicable, is the statistical analysis and its interpretation appropriate?

Yes

Are all the source data underlying the results available to ensure full reproducibility?

Yes

Is the study design appropriate and is the work technically sound?

Yes

Are the conclusions drawn adequately supported by the results?

Yes

Are sufficient details of methods and analysis provided to allow replication by others?

Yes

Reviewer Expertise:

otolaryngology, epidimiology, PCV's

I confirm that I have read this submission and believe that I have an appropriate level of expertise to confirm that it is of an acceptable scientific standard, however I have significant reservations, as outlined above.

# Reviewer report: Reviewer response for version 1

**DOI:** 10.21956/wellcomeopenres.21354.r82660

**Published:** 2024-06-12

**Authors:** Dorcas M Kamuya

**Affiliations:** 1 Kenya Medical Research Institute (KEMRI) - Wellcome Trust Research Programme, Kilifi, Kenya

Controlled Human Infection studies are increasingly being introduced in LMICs including in Africa. They raise a plethora of social, ethical, scientific and cultural issues. A growing body of literature is helping to unpack these issues; the paper therefore feeds into this body of work. The area of recruitment strategies has perhaps not been well addressed, this paper helps fill this gap. There are a few areas I raise below that could strengthen the paper.

 1. References are few, yet there is a body of work on this area across Africa, that can be drawn up on.

 2. First paragraph 2nd sentence - could you provide some examples and references of how HIS studies have led to prevention and treatment of infectious diseases - these outcomes seems not to be directly related to CHIM, but I could be wrong

 3. 2nd paragraph - Malawi, Kenya etc - also include what contribution such CHIM studies conducted in African countries have made (as you did in the previous sentence for HIC).

 4. Objectives these are not clearly defined. what was the objective of the present study (ie not the objective of this paper). Also, could add the overall research question of the study.

 5. add information on the study site and within that include the description of the CHIM. Please also note that the section on trial design is not really part of qualitive method and would fit well under the study site description.

 5. Under methods - include information on how the data for this paper were collected, analysed etc. The methods that are currently described are those of the recruitment strategies - these should be provided either under results section (where you then start by describing the different recruitment strategies used for the CHIMN study) or - depending on how the info is framed - can be described under study site - where info about the CHIM study would be included. Also would be great to include information in why the CHIM study targeted University students and not the general population  

 6. It is very interesting that the snowballing was by those that attended the information giving sessions - where any information materials given to them to share within the populations they come from? In case not, was there an attempt to find out what information was shared (in our setting in Kenya, we realised that most info shared at that stage were about the health benefits, the compensation amounts and the risks of the study, with a greater emphasis on financial compensation  - did similar issues also arise in your setting?). This is really important as later under results, it is reported that 71.9% of those that were screened 'indicated that they were motivated to join through snowball recruitment'. What information were they given to motivate them to join the study? also what does motivation in this instance mean - noting also that it has been used under sensitization as well? is it motivation or encouraged?

 7. The discussion section of the paper needs strengthening (major revisions). It would be important to situate the recruitment strategies within wider debates of recruitment in clinical trials (if indeed none of the strategies have been discussed within CHIM studies). Interrogate the strategies - their strengths and weaknesses, and position such critique within for example some of the well known ethical principles and frameworks including for example = that of relational autonomy, networks and value of social groups (pros and cons). Also, you can be reflective here and suggest what this means for similar studies in Malawi and elsewhere.

 8. As suggested in point 5 - this paper can be strengthened by integrating the recruitment strategies descriptions with the quantitative information provided under results. 

 9. could the authors whether the  ethics approvals quoted are for the main CHIM study or are for this descriptive mixed methods, alternatively could include the approval for this specific study 

 10. would be great to include the link to the Youtube video about CHIM. Is it translated to English for wider audience? Also include a sample consent form as an attachment.

 Finally, with strengthening of this paper as suggested above, it would make an important about recruitment strategies for CHIM in LMICs, and provide a critical reflection for other sites to consider too.

Is the work clearly and accurately presented and does it cite the current literature?

Partly

If applicable, is the statistical analysis and its interpretation appropriate?

Not applicable

Are all the source data underlying the results available to ensure full reproducibility?

Yes

Is the study design appropriate and is the work technically sound?

Partly

Are the conclusions drawn adequately supported by the results?

Partly

Are sufficient details of methods and analysis provided to allow replication by others?

No

Reviewer Expertise:

Bioethics, ethical issues in Human Infection studies, community engagement

I confirm that I have read this submission and believe that I have an appropriate level of expertise to confirm that it is of an acceptable scientific standard, however I have significant reservations, as outlined above.

# Response

**Published:** 2025-02-19

**Authors:** Edna Nsomba

**Affiliations:** Malawi Liverpool Wellcome Trust, Malawi

1.References are few, yet there is a body of work on this area across Africa, that can be drawn up onReferences are few, yet there is a body of work on this area across Africa, that can be drawn up on

- Thank you very much. We have reviewed more literature on this and what we are finding is this there are not many CHIM studies except for the two added.Please refer to the two research papers on Malaria CHIM study in Kenya and the first at risk population CHIM study conducted in Malawi have been added on page 7.If you have any references in mind, we will be happy to add them.

2. First paragraph 2nd sentence - could you provide some examples and references of how HIS studies have led to prevention and treatment of infectious diseases - these outcomes seems not to be directly related to CHIM, but I could be wrong

- A reference on how HIS studies have led to prevention and treatment of infectious diseases has been added on page 7.

3. 2nd paragraph - Malawi, Kenya etc - also include what contribution such CHIM studies conducted in African countries have made (as you did in the previous sentence for HIC).

- Please refer to references on page 6

4. Objectives these are not clearly defined. what was the objective of the present study (i.e. not the objective of this paper). Also, could add the overall research question of the study.

- We acknowledge this.Please refer to page 8 of the manuscript.

5. add information on the study site and within that include the description of the CHIM. Please also note that the section on trial design is not really part of qualitive method and would fit well under the study site description.

- Thank you for pointing this out.The section has been revised. Please refer to a description made on page 8.

6. Under methods - include information on how the data for this paper were collected, analysed etc. The methods that are currently described are those of the recruitment strategies - these should be provided either under results section (where you then start by describing the different recruitment strategies used for the CHIM study) or - depending on how the info is framed - can be described under study site - where info about the CHIM study would be included. Also would be great to include information in why the CHIM study targeted University students and not the general population 

- Thank you for pointing this out.Please refer to page 8 on data collection and analysis.

7. It is very interesting that the snowballing was by those that attended the information giving sessions - where any information materials given to them to share within the populations they come from? In case not, was there an attempt to find out what information was shared (in our setting in Kenya, we realised that most info shared at that stage were about the health benefits, the compensation amounts and the risks of the study, with a greater emphasis on financial compensation - did similar issues also arise in your setting?). This is really important as later under results, it is reported that 71.9% of those that were screened 'indicated that they were motivated to join through snowball recruitment'. What information were they given to motivate them to join the study? also what does motivation in this instance mean - noting also that it has been used under sensitization as well? is it motivation or encouraged?

- During our community engagement sessions in colleges, we distributed participants information sheets (PIS) in both English and local language (Chichewa). Potential participants could also share these PIS to their families and friends to read. We would like to acknowledge that we did not find out from the participants what kind of information they got from their colleagues. This has been included in the discussion as a limitation.

8. The discussion section of the paper needs strengthening (major revisions). It would be important to situate the recruitment strategies within wider debates of recruitment in clinical trials (if indeed none of the strategies have been discussed within CHIM studies). Interrogate the strategies - their strengths and weaknesses, and position such critique within for example some of the well known ethical principles and frameworks including for example = that of relational autonomy, networks and value of social groups (pros and cons). Also, you can be reflective here and suggest what this means for similar studies in Malawi and elsewhere.

- This is acknowledged and we have done the revisions.Please refer to page 13 for a revised discussion version.

9.As suggested in point 5 - this paper can be strengthened by integrating the recruitment strategies descriptions with the quantitative information provided under results. 

- This is noted. Please refer to first paragraph of the discussion section for an addition of the quantitative information.

10.could the authors whether the ethics approvals quoted are for the main CHIM study or are for this descriptive mixed methods, alternatively could include the approval for this specific study.

- The approvals quoted are for the main CHIM study because this study did not have a separate protocol.

11. would be great to include the link to the Youtube video about CHIM. Is it translated to English for wider audience? Also include a sample consent form as an attachment.

 Finally, with strengthening of this paper as suggested above, it would make an important about recruitment strategies for CHIM in LMICs, and provide a critical reflection for other sites to consider too.

- The link to the tube video and a sample consent form are attached.The you tube video is in English and currently translation to Chichewa (our local language) is underway through our science and communication department.
